# Durability of Polymer-Modified Reclaimed Asphalt Mixtures Rejuvenated with Simulated Waste Cooking Oils from Palm, Soy, Olive, and Rice Oils

**DOI:** 10.3390/polym18070833

**Published:** 2026-03-28

**Authors:** Kyungnam Kim, Lee Ho Joung, PARK Jin Woo, Tri Ho Minh Le

**Affiliations:** 1 Pavement Research Division, Korea Expressway Corporation Research Institute, Dongtansunhwan-daero 17-gil, Hwaseong-si 18489, Gyeonggi-do, Republic of Korea; kkn248@ex.co.kr; 2 Education & Public Relations Department, Korea Road Association (KROAD), 8F, 26 Wiryeseoil-ro, Sujeong-gu, Seongnam-si 13647, Gyeonggi-do, Republic of Korea; hjlee@kroad.or.kr (L.H.J.); pjw@kroad.or.kr (P.J.W.); 3 Faculty of Civil Engineering, Nguyen Tat Thanh University, 300A Nguyen Tat Thanh Street, District 4, Ho Chi Minh City 70000, Vietnam

**Keywords:** simulated waste cooking oil, polymer-modified RAP, rejuvenator dosage, Hamburg wheel tracking, moisture susceptibility, cracking resistance

## Abstract

Reclaimed asphalt pavement (RAP) from polymer-modified asphalt pavements often contains a recovered binder that is stiff and brittle, which reduces workability and increases durability risk. Waste cooking oil (WCO) is a promising circular rejuvenator, but its effectiveness remains inconsistent because oil source and degradation state are often not well controlled, particularly in polymer-modified RAP systems. This study introduced a controlled simulated WCO approach and compared four oil sources (Palm, Soy, Olive, and Rice) as rejuvenators for recovered RAP binder and RAP mixtures. Simulated oils were added at 4% and 8% by mass of recovered RAP binder. The simulated WCOs produced clear dosage-dependent softening of the recovered binder. Penetration increased, while softening point and rotational viscosity decreased, indicating partial restoration of binder mobility and improved workability. At the mixture level, the 4% dosage provided the most balanced performance, improving moisture resistance and reducing Cantabro loss compared with the control mixture. Specifically, tensile strength ratio (TSR) increased from 75% to 80.9–83.7%, while Cantabro loss decreased from 19.8% to 13.2–14.6%, showing better cohesion and resistance to particle loss. However, Hamburg wheel tracking (HWT) results revealed strong oil-source dependence, with Soy showing the lowest rut depth and Olive the highest, indicating that excessive softening can reduce deformation resistance. The results demonstrate that controlled simulated WCO can support practical oil-source selection for polymer-modified RAP mixtures. A moderate dosage is more effective because it improves binder restoration and mixture durability without causing excessive softening, while rutting verification remains essential before field application.

## 1. Introduction

The use of RAP is one of the most practical strategies for reducing the cost and environmental burden of asphalt pavement construction [1]. However, the binder recovered from RAP is inherently aged by long-term oxidative hardening, and this issue becomes more critical when RAP is sourced from polymer-modified asphalt pavements. In polymer-modified systems, aging affects not only the base binder but also the polymer-rich phase and the overall binder compatibility [2]. As a result, RAP mixtures produced without proper binder restoration often exhibit reduced workability during production, higher brittleness, and a higher risk of moisture and cracking-related damage during service. These limitations are a major barrier to increasing RAP usage in surface courses where durability requirements are strict [3].

Rejuvenators are commonly applied to restore the rheological balance of aged binders by replenishing the lighter fractions and improving binder mobility [4]. Among available rejuvenators, waste cooking oil has received substantial attention because it is widely available, low-cost, and circular economy-oriented.

Waste cooking oil and other bio-oils are increasingly used as rejuvenators for aged binders because they replenish lighter fractions and improve binder mobility, but the literature repeatedly stresses that oil chemistry and variability control govern repeatability. Gul et al. [5] summarized the state of the art and emphasized the need to balance cracking benefits against potential rutting penalties. Zhong et al. [6] showed that linking WCO physicochemical indices to reclaimed binder properties can provide strong prediction with R^2^ above 0.85, supporting standardized selection rather than trial and error. Li et al. [7] combined rheology with GPC and AFM and reported substantial stiffness reductions and improved low-temperature indicators after waste edible oil addition, consistent with a restoration mechanism dominated by physical blending and colloidal restructuring. Comparative studies also confirm strong oil-type dependence: five bio-oils can deliver different high-temperature penalties and low-temperature benefits, while Yuan et al. [8] reported large stiffness reductions using pine needle oil and supported compatibility improvements using molecular dynamics. Zheng et al. [9] proposed conditioning high-acid used edible oil and recommended an optimum dosage window around 4.4 to 7%, again highlighting that process control is essential.

Recent studies have expanded the use of WCO and other bio-based rejuvenators for RAP binders and mixtures. For example, Gooi et al. [10], Ye et al. [11], and Fei et al. [12] investigated Palm-oil- or WCO-based rejuvenation approaches, while Melo Neto et al. [13], Xiong et al. [14], Alizadeh et al. [15], Hutabarat et al. [16], Lee et al. [17], and Guodong et al. [18] examined other bio-oil or composite rejuvenator systems at binder and mixture levels. In parallel, Zhang et al. [19], Tushar et al. [20], and Islam and Hossain [21] highlighted the importance of rejuvenator selection in high-RAP, SBS-modified, and sustainability-oriented applications, whereas Gul et al. [5] provided a recent review of WCO as a green rejuvenator for RAP. Overall, these studies show that rejuvenator source, processing method, and dosage strongly influence the final performance.

At the mixture and sustainability scale, recent work converges on performance plus environmental evaluation rather than binder softening alone. Hu et al. [22] reported that waste oil-activated rubberized foamed asphalt in cold recycling can reduce manufacturing temperature by 15 °C and deliver large life cycle reductions in greenhouse gas emissions and energy demand. Zhao et al. [23] showed that low-dosage epoxidized soybean oil in cold central plant recycling can achieve moisture resistance near 88% TSR together with meaningful carbon reduction. Wang et al. [24] reported very large Global Warming Potential reductions when combining waste engine oil residue with crumb rubber, while also noting environmental tradeoffs that must be tracked. Yang et al. [1] extended recycling beyond the binder by using bio-oil-based separation and surface treatment to enable full RAP aggregate recycling with performance effects at the mixture level. Hassanjani et al. [25] framed high recycled contents using a balanced mix design approach and reported simultaneous improvements in cracking indices with reduced emissions and costs, and Nagy et al. [26] reinforced this direction using LCA plus multi-criteria decision frameworks. Pasciucco et al. [27] and Qian Wang et al. [9] further support the broader circular economy case by showing that controlled WCO upgrading routes can deliver sizable CO_2_ reductions and stable multi-cycle catalytic performance in downstream products.

Nevertheless, waste cooking oil is not a single material. Its chemical composition and degradation state depend strongly on the original oil source and the cooking history, which leads to large variability in viscosity, acidity, and interaction potential with oxidized binder fractions [28,29,30,31]. In many published studies, this variability is either uncontrolled or treated as secondary, and performance is often reported for one oil type without a systematic comparison [27,28,30,32]. As a consequence, it remains difficult to translate laboratory findings into robust selection guidance for agencies and contractors, particularly for RAP sourced from polymer-modified pavements, where excessive softening can quickly translate into rutting penalties.

Another practical gap is that many studies emphasize binder softening indicators alone, while mixture level durability is governed by a balance among rutting resistance, moisture resistance, and cracking tolerance. A rejuvenator that improves cracking resistance may reduce rutting stability, and a rejuvenator that improves workability does not always improve resistance to moisture-induced damage [32]. For polymer-modified RAP, this balance is even more sensitive because the binder system may show strong stress sensitivity and different damage evolution under combined water and loading. Therefore, a binder to a mixture framework that compares rejuvenator candidates under consistent simulated waste oil conditions is needed.

The novelty of this study lies in establishing a controlled simulated waste cooking oil framework to isolate the effect of oil source on the rejuvenation of polymer-modified RAP systems, where both aged asphalt and degraded polymer structure influence performance. Unlike many previous studies using field-collected waste oils with poorly controlled histories, this work compares Palm, Soy, Olive, and Rice oils under the same thermal conditioning protocol, allowing a more reproducible source-based evaluation. More importantly, the study does not stop at binder softening, but builds a binder-to-mixture performance chain that shows how oil-dependent changes in viscosity, acidity, rheological softening, and stress sensitivity are reflected in moisture resistance, raveling resistance, cracking tolerance, and rutting behavior. In this way, the study provides both a clearer scientific basis and a practical screening framework for selecting rejuvenator type and dosage in RAP mixtures produced from polymer-modified pavements.

In this study, simulated waste cooking oils were generated from four widely used edible oil sources, Palm, Soy, Olive, and Rice, using a controlled thermal-conditioning procedure to reduce the uncertainty associated with field-collected oils. The oils were characterized using simple practical indices, namely viscosity at 40 °C and acid value, then used to rejuvenate a recovered RAP binder from a polymer-modified pavement at two dosages (4% and 8% by mass of RAP binder). Binder screening included penetration, softening point, rotational viscosity, frequency sweep master curve stiffness, and MSCR strain response at 0.1 and 3.2 kPa to capture both softening and stress-dependent deformation tendency. Mixture performance was evaluated using moisture-conditioned indirect tensile strength (ITS) and tensile strength ratio (TSR), Cantabro loss, overlay test (OT) cracking index, semicircular bending (SCB), load–displacement response and fracture energy, and HWT under submerged conditions to directly assess rutting and moisture-related durability.

## 2. Materials and Methods

### 2.1. Materials

#### 2.1.1. Binder

This study considered two binder sources: an original polymer-modified asphalt binder (PMAB) representative of the pavement binder system, and the binder recovered from RAP collected from a polymer-modified asphalt pavement (recovered PMAB–RAP binder) [33,34]. The comparison is intended to quantify the extent of hardening in the recovered RAP binder relative to the original polymer-modified binder, and to justify the need for rejuvenation treatment prior to mixture performance evaluation. The materials used in this research were provided through collaboration with asphalt construction companies in Vietnam and South Korea.

In general, the recovered PMAB–RAP binder exhibits substantially higher stiffness and reduced workability compared with the original PMAB, as shown in Table 1. This is reflected by lower penetration and higher softening point, indicating oxidative aging and loss of flowability after field service. Dynamic viscosity at 135 °C and 165 °C further confirms the reduced workability of the recovered binder, which can affect mixing, coating, and compaction quality when RAP is incorporated. These baseline differences between the original PMAB and recovered RAP binder provide the material basis for the subsequent binder modification and mixture performance evaluation presented in this manuscript.

#### 2.1.2. Aggregate Mix

##### Original Aggregate

Crushed basalt aggregates sourced from a local quarry in Southern Vietnam were used as the virgin aggregate for all mixture preparations. The aggregate was oven-dried and separated into individual size fractions by sieve analysis following ASTM C136 [38] to produce the target dense graded aggregate structure. Key physical properties are summarized in Table 2. The virgin coarse aggregate had a bulk specific gravity (SSD) of approximately 2.85 and water absorption of 0.9%, while the virgin fine aggregate had a bulk specific gravity (SSD) of 2.80 and water absorption of 1.3%. The Los Angeles abrasion loss of the coarse fraction was approximately 20%, indicating adequate resistance to abrasion for surface course applications, while the flakiness index was approximately 16%, supporting a stable aggregate skeleton and interlock performance.

##### RAP Aggregate

**Figure 1:**
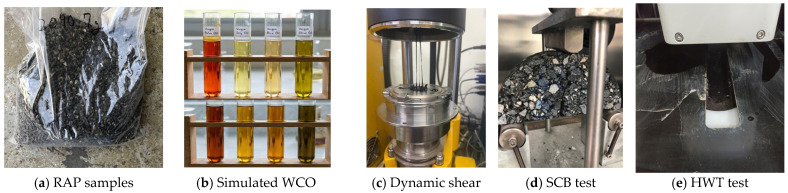
Photographs of materials and test setups: (**a**) RAP samples, (**b**) simulated WCO, (**c**) dynamic shear rheometer (DSR) test, (**d**) semi-circular bending (SCB) test, and (**e**) HWT test.

RAP was collected from a polymer-modified pavement surface layer and processed by crushing and screening to obtain a representative laboratory RAP stockpile (see Figure 1a). The RAP content in the mixture was fixed at 25% by total mixture mass. For reference, the aggregate component of RAP (after binder removal and cleaning) exhibited a bulk specific gravity of approximately 2.78 and water absorption of 1.0%, which are comparable to basalt aggregates but reflect the presence of adhered aged binder film and surface texture changes associated with reclaimed materials. The Los Angeles abrasion loss and flakiness index of the recovered RAP aggregate were approximately 24% and 18%, respectively, indicating slightly lower mechanical durability than the virgin basalt, as commonly observed for reclaimed aggregates. These RAP aggregate properties were considered during mixture design and volumetric control, while the virgin aggregate blend and target gradation were kept constant across mixtures to isolate the effect of simulated WCO rejuvenation.

### 2.2. Additive

Four edible oils were used as rejuvenator additives: Palm, Soy, Olive, and Rice oils (Table 3; Figure 1b). To generate repeatable simulated waste cooking oils, each fresh oil was thermally conditioned at 180 ± 2 °C in an open 1 L borosilicate glass beaker (inner diameter ~120 mm) containing 500 g of oil (fill depth ~45–50 mm) under ambient atmospheric exposure for 6 h with no stirring to standardize air–oil contact and oxidation severity, representing typical frying temperatures. The oils were thermally conditioned at 180 ± 2 °C for 6 h to generate a controlled and repeatable degradation state prior to use as simulated WCO. This temperature was selected because it falls within the typical range of deep-frying conditions, while the 6 h duration was adopted to induce clear thermal degradation under a standardized laboratory protocol. The purpose of this treatment was not to reproduce a fixed number of real frying cycles, since actual waste cooking oil degradation depends on oil type, frying practice, food residue, moisture, and reheating history, but rather to establish a consistent simulated-WCO condition for comparing oil-source effects in the subsequent binder and mixture evaluations. Temperature was monitored using a thermocouple immersed in the oil. After conditioning, the oil was cooled to room temperature and filtered through a 0.45 mm stainless-steel mesh to remove insoluble residues before use.

Oils were characterized before and after conditioning by (i) dynamic viscosity at 40 °C and (ii) acid value, as indices relevant to diffusion/flowability and thermal degradation, respectively. Conditioning increased acid value and changed viscosity, while preserving oil-type trends: Olive and Soy showed lower viscosity and higher acid value, Palm showed the highest viscosity and lowest acid value, and Rice was intermediate, consistent with the relative softening trends observed in binder tests.

**Figure 2:**
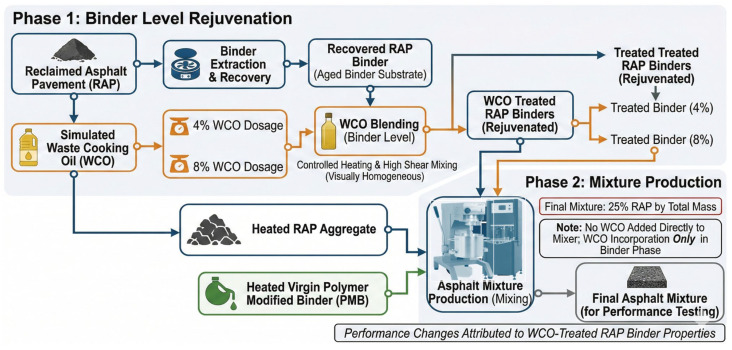
General research flowchart.

In general, the experimental workflow was executed in two systematic phases, as shown in Figure 2. In Phase 1 (Binder Level Rejuvenation), aged binder extracted from reclaimed asphalt pavement (RAP) was blended with simulated WCO at 4% and 8% dosages. This process utilized controlled heating and high-shear mixing to ensure a visually homogeneous, rejuvenated RAP binder. In Phase 2 (Mixture Production), the rejuvenated binders were combined with heated RAP aggregate and heated virgin polymer-modified binder (PMAB) to produce a final asphalt mixture containing 25% total RAP content. WCO incorporation was strictly limited to the binder phase to ensure that subsequent performance changes were directly attributable to the properties of the treated RAP binders.

The simulated WCO dosages of 4% and 8% by mass of recovered RAP binder were selected based on previous studies on oil-based rejuvenators [31,32,42,43] and preliminary laboratory screening conducted before the main test program. The 4% dosage was chosen as a moderate level expected to provide partial restoration of the aged binder without excessive softening, whereas the 8% dosage was selected as a higher level to evaluate the effect of stronger softening and to assess the potential over-softening risk. By considering these two dosage levels, the study aimed to compare the performance of a more balanced rejuvenation condition with that of a relatively aggressive rejuvenation condition.

### 2.3. Binder Preparation and Blending Procedure

All binders were prepared using the recovered RAP binder as the base material, followed by incorporation of simulated WCO according to the target dosage levels. Two simulated WCO dosages were evaluated, 4% and 8%, defined as wt.% relative to the recovered RAP binder mass, and four oil sources were considered (Palm, Soy, Olive, and Rice). Table 4 summarizes the binder compositions used in this study. The control binder consisted of recovered RAP binder without simulated WCO (Control). For modified binders, simulated WCO was introduced at either 4% or 8% by mass of recovered RAP binder, and the total binder content in the mixture was held constant at 5.2%, which includes virgin binder + RAP binder + simulated WCO (i.e., simulated WCO partially replaces the added virgin binder to maintain the same total binder content). The total asphalt binder content of 5.2 wt.% was selected based on previous studies [33,34,44] and preliminary laboratory trial mixture experiments conducted before the main test program. These preliminary trials were used to identify a practical binder content for the target dense-graded mixture containing 25 wt.% RAP and to ensure workable mixing and specimen preparation under the selected gradation condition. The resulting binders are denoted as Palm4%, Soy4%, Olive4%, Rice4%, and Palm8%, Soy8%, Olive8%, Rice8%.

For binder preparation, the recovered RAP binder was heated to 160 ± 5 °C to achieve adequate fluidity. Simulated WCO was preheated to 135 ± 5 °C and added gradually to the RAP binder to minimize thermal shock. Blending was performed in a 1 L metal can using an overhead mechanical stirrer equipped with a four-blade propeller impeller (diameter 50 mm, positioned at mid-depth), with a total binder batch of 500 g. The mixture was stirred at 600 rpm for 20 min while maintaining 160 ± 5 °C (temperature monitored by a probe immersed in the binder); the vessel was partially covered to limit volatilization and heat loss. After WCO incorporation, the modified binder was conditioned for an additional 30 min at 160 °C under gentle stirring to promote diffusion and stabilize the blend prior to testing.

Binder characterization was conducted on unaged samples. Where short-term conditioning was required for consistency with mixture production, RTFO aging was applied following ASTM D2872 as a conditioning step to represent the thermal history associated with asphalt mixing and short-term handling, rather than to evaluate long-term aging resistance.

For clarity and consistency, the term “simulated WCO” refers to the thermally conditioned oil used in this study, “simulated WCO-modified binder” refers to the recovered RAP binder blended with simulated WCO, and “simulated WCO mixture” refers to the asphalt mixture produced using the corresponding modified binder.

### 2.4. Binder Testing Methods

A binder testing program was conducted to quantify the effects of simulated WCO type and dosage on the consistency, workability, and rutting-related rheological response of the recovered RAP binder. All tests followed relevant ASTM standards.

Penetration was measured at 25 °C in accordance with ASTM D5 [35], and the softening point was determined by the Ring-and-Ball method according to ASTM D36 [36], following a procedure similar to that used by Langa et al. [45]. Rotational viscosity was measured following ASTM D4402 at 135 °C and 165 °C to evaluate high-temperature workability.

Rheological characterization was performed using a DSR in accordance with ASTM D7175 [46].

Frequency sweeps were performed at angular frequencies ω=0.1–100 rad/s at multiple temperatures to obtain the complex modulus magnitude $\left|G^{*}\right|$. The data were shifted to a reference temperature $T_{ref}$ using time–temperature superposition to construct $\left|G^{*}\right|$ master curves. The reduced angular frequency was defined as $\omega_{r}=a_{T}\omega$, where $a_{T}$ is the temperature shift factor. Shift factors were determined by minimizing the mismatch between adjacent isotherms and fitted using the Williams–Landel–Ferry (WLF) equation:

(1) $${log}_{10}(a_{T})=-\frac{C_{1}\;(T-T_{ref})}{C_{2}+(T-T_{ref})}$$

The master curve was then fitted using a sigmoidal model:

(2) $${log}_{10}|G^{*}|=\delta +\frac{\alpha }{1+exp\left(\beta +\gamma \;{log}_{10}(\omega_{r})\right)}$$

where α, β, γ, and δ are fitting parameters.

Multiple stress creep recovery tests were conducted in accordance with ASTM D7405 [47]. MSCR loading was applied at 0.1 kPa and 3.2 kPa, using 1 s creep and 9 s recovery per cycle for 10 cycles at each stress level. The strain response was used to compare stress sensitivity and deformation tendency among the control binder and the simulated WCO-modified binder.

### 2.5. Asphalt Mixture Preparation

Virgin aggregates were oven dried at 110 ± 5 °C and preheated to the designated mixing temperature. RAP materials were dried at 60 to 70 °C to minimize additional oxidative aging while removing moisture. Virgin and RAP aggregates were combined in the specified proportions and dry-mixed for 60 to 90 s to promote temperature uniformity prior to binder addition. The prepared binder (virgin binder with or without simulated WCO) was introduced gradually under continuous mechanical mixing, and wet mixing was performed for 120 to 180 s to ensure uniform coating [33]. Mixture production temperature was maintained at 160 ± 5 °C, and compaction temperature was set at 150 ± 5 °C, consistent with HMA practice and the viscosity levels reported in Section 3.1.

Compaction was performed using a Superpave gyratory compactor at N_design = 75 gyrations, targeting 4.0 ± 0.5% air voids. After compaction, specimens were cooled at room temperature for 24 h and then cut or conditioned to meet the dimensional requirements for subsequent testing.

### 2.6. Mixture Testing Methods

A mixture-level testing program was conducted to evaluate moisture susceptibility, raveling resistance, rutting and moisture-related damage, and cracking resistance of RAP mixtures incorporating simulated WCO. Unless otherwise noted, specimens were stored at (25 ± 2) °C for 24 h after compaction prior to testing.

Indirect tensile strength (ITS) was measured according to ASTM D6931 [48] and moisture susceptibility was evaluated using the tensile strength ratio (TSR) following AASHTO T283 [49]. Gyratory-compacted cylindrical specimens of 100 mm diameter and 63.5 mm height were tested at 25 °C using a constant loading rate of 50 mm/min. TSR was computed as the ratio of the average ITS of conditioned specimens to unconditioned specimens. Conditioned specimens were subjected to one freeze–thaw cycle per AASHTO T283, while unconditioned specimens were kept dry at 25 °C.

Raveling resistance was evaluated using the Cantabro loss test in accordance with AASHTO TP 108-14 [50]. Gyratory-compacted specimens of 100 mm diameter were tested at room temperature in the Los Angeles abrasion drum for 300 revolutions without steel balls. Cantabro loss was calculated as the percentage mass loss relative to the initial dry mass.

Reflective cracking resistance was evaluated using the overlay test according to Tex 248-F. Beam specimens (150 mm × 75 mm × 38 mm) were conditioned at 25 °C and subjected to repeated opening and closing displacement of 0.025 in (0.635 mm) at the standard cycling rate specified in Tex 248-F [51], and the retained load index versus cycles was used for comparison. Low-temperature fracture behavior was evaluated using the semi-circular bending (see Figure 1d) test following ASTM D8044 [52] at 0 °C, using semi-circular specimens of 150 mm diameter and 50 mm thickness with a notch depth of 15 mm. Load–displacement responses were used to compute fracture energy and post-peak behavior.

Rutting and stripping susceptibility were assessed using the Hamburg wheel tracking test following AASHTO T324 [53] (see Figure 1e). Slab specimens were prepared to the standard Hamburg dimensions (260 mm × 320 mm × 50 mm), submerged in a 50 °C water bath, and tested to 20,000 wheel passes while rut depth was recorded continuously. Rut depth progression and the onset of stripping-related acceleration were used for comparative evaluation.

Overlay, SCB, and Hamburg were conducted for the Control and 4% mixtures to focus on the dosage that provided the best balance in screening tests.

## 3. Results and Discussions

### 3.1. Binder Testing Results

#### 3.1.1. Penetration and Softening Test Results

**Figure 3:**
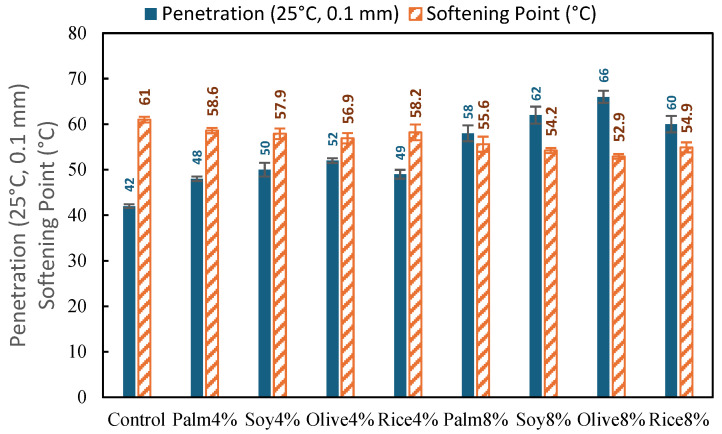
Penetration and softening point of control RAP binder and simulated WCO-modified binder (4% and 8%).

Figure 3 summarizes the effects of simulated WCO type and dosage on the basic consistency indices of the control RAP binder, which is expected to be stiff due to oxidative aging and the polymer-modified origin of the reclaimed material. The control binder exhibited a penetration of 42 (0.1 mm) and a softening point of 61.0 °C, indicating a high consistency and reduced flowability typical of aged RAP binders.

Across all simulated WCO types, adding WCO increased penetration and reduced softening point, confirming a clear rejuvenation effect driven by plasticization. At the 4% dosage, penetration increased from 48 to 52 (0.1 mm), which corresponds to approximately 14 to 24% higher than the Control, while the softening point decreased from 56.9 to 58.6 °C, representing a drop from 2.4 to 4.1 °C, or roughly 4 to 7%. These shifts are consistent with diffusion of lighter oil fractions into the aged binder matrix, which partially restores a maltene-like phase and increases molecular mobility. The oil source effect is also evident at 4%. Olive4% produced the strongest softening (penetration 52, softening point 56.9 °C), whereas Palm4% showed the mildest response (penetration 48, softening point 58.6 °C), with Soy4% and Rice4% remaining intermediate. This ranking can be linked to differences in simulated WCO properties after thermal conditioning. Oils with lower viscosity and higher acid value tend to diffuse more readily and interact more effectively with oxidized binder fractions, leading to larger penetration increases and greater softening point reductions at the same dosage. In this study, the example oil property trend is consistent with that interpretation, with Olive and Soy showing lower viscosity and higher acid value than Palm, while Rice remains intermediate. This is in agreement with Zhong et al. [6] and with the broader bio-oil rejuvenation literature reporting binder plasticization and dosage-dependent softening of aged asphalt binders

When the dosage increased to 8%, the consistency changes became much larger. Penetration rose from 58 to 66 (0.1 mm), which is about 38 to 57% higher than the Control, while softening point dropped from 52.9 to 55.6 °C, corresponding to a decrease of 5.4 to 8.1 °C, about 9 to 13%. The strongest softening again occurred for Olive8% (penetration 66, softening point 52.9 °C), followed by Soy8% and Rice8%, while Palm8% remained the least aggressive. From a performance standpoint, this stronger shift at 8% is a trade-off. It indicates more effective restoration of binder flexibility, which is favorable for cracking resistance, but it also implies reduced high-temperature consistency and elastic response, which can increase rutting susceptibility. This point is particularly relevant for RAP derived from polymer-modified pavements, where maintaining high temperature stability and phase compatibility remains important.

#### 3.1.2. Dynamic Viscosity Test Results

**Figure 4:**
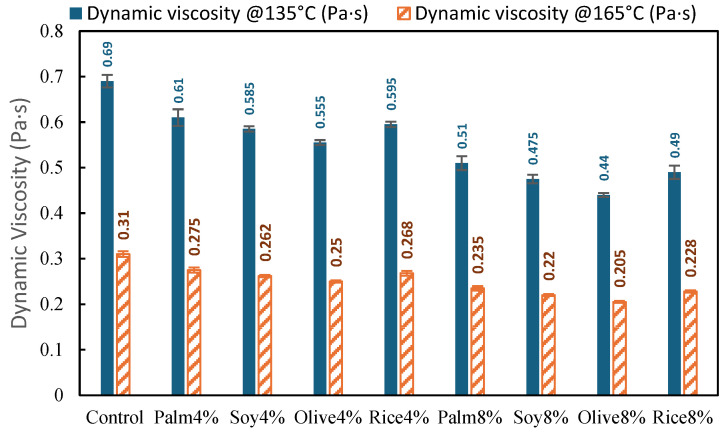
Dynamic viscosity of control RAP binder and simulated WCO-modified binder at 135 °C and 165 °C.

Figure 4 presents the dynamic viscosity results of the control RAP binder and the simulated WCO-treated binders at 135 °C and 165 °C. The control binder showed viscosities of 0.69 Pa·s at 135 °C and 0.31 Pa·s at 165 °C, which is consistent with the high stiffness and reduced workability typically associated with aged RAP binders. As expected, viscosity decreased with increasing temperature for all binders, reflecting thermally activated flow behavior.

Across all simulated WCO types, adding WCO reduced viscosity at both temperatures, confirming improved binder workability. At the 4% dosage, viscosity at 135 °C decreased from 0.69 Pa·s to 0.555 to 0.61 Pa·s, corresponding to a reduction of approximately 12 to 20% relative to the Control. The lowest value within the 4% group was observed for Olive4% (0.555 Pa·s, about 19.6% lower than control), while Palm4% (0.61 Pa·s, about 11.6% lower) showed the mildest reduction. Soy4% (0.585 Pa·s) and Rice4% (0.595 Pa·s) remained intermediate. A similar trend was observed at 165 °C, where viscosities decreased from 0.31 Pa·s (control) to 0.25 to 0.275 Pa·s for the 4% binders, representing about 11 to 19% reduction. Again, Olive4% (0.25 Pa·s) showed the largest decrease, and Palm4% (0.275 Pa·s) the smallest. This is in agreement with Li et al. [7], who similarly demonstrated that the addition of waste edible and cooking oils restores the viscosity of aged asphalt and successfully enhances the overall workability and molecular flow behavior of the binder.

When the dosage increased to 8%, viscosity reductions became more pronounced. At 135 °C, viscosity decreased from 0.44 to 0.51 Pa·s, equivalent to approximately 26 to 36% lower than the Control. The strongest effect was observed for Olive8% (0.44 Pa·s, about 36.2% lower), followed by Soy8% (0.475 Pa·s, about 31.2% lower) and Rice8% (0.49 Pa·s, about 29.0% lower), while Palm8% (0.51 Pa·s, about 26.1% lower) remained the least aggressive. At 165 °C, viscosities dropped from 0.205 to 0.235 Pa·s, representing about a 24 to 34% reduction relative to the control, with the same oil ranking. Figure 4 confirms a clear dosage-dependent improvement in binder flow behavior, and the oil-type ranking is consistent with the penetration and softening point trends in Figure 3.

#### 3.1.3. MSCR Test Results

**Figure 5:**
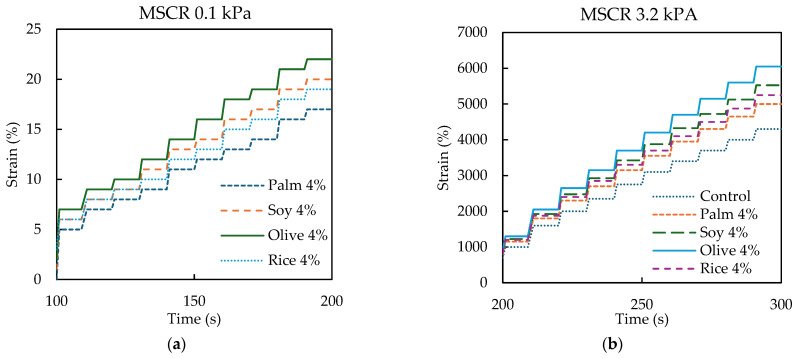
MSCR strain response of control RAP binder and simulated WCO-modified binder at (**a**) 0.1 kPa and (**b**) 3.2 kPa.

Figure 5a,b presents the MSCR strain accumulation, reported here as % strain, under low stress (0.1 kPa) and high stress (3.2 kPa). In both figures, the curves show the expected stepwise increase because each MSCR cycle consists of a short creep loading period followed by a recovery period, so the strain rises during loading and then partially recovers before the next cycle begins. The remaining strain after each cycle, therefore, accumulates progressively with time.

In Figure 5a (0.1 kPa), the percent strain levels are relatively small and the separation among the 4% simulated WCO binders is moderate. By the end of the test window (200 s), Palm4% reaches 17%, Rice4% reaches 19%, Soy4% reaches 20%, and Olive4% reaches 22%. This ordering suggests that Olive4% produces the highest strain accumulation, while Palm4% produces the lowest, within the 4% dosage group. A practical interpretation is that oils that produce stronger softening in basic consistency and viscosity tests also allow greater strain accumulation even under low stress, likely due to reduced binder stiffness and reduced resistance to delayed deformation [54].

In Figure 5b (3.2 kPa), the stress sensitivity becomes much more pronounced and the differences among binders are amplified. The control binder exhibits the lowest strain accumulation throughout the test, reaching 4300% at 300 s. All 4% simulated WCO binders show higher accumulated strains, reaching 5000% (Palm4%), 5250% (Rice4%), 5525% (Soy4%), and 6050% (Olive4%) at 300 s. Relative to the control, these correspond to approximately +16% (Palm4%), +22% (Rice4%), +29% (Soy4%), and +41% (Olive4%). The oil ranking remains consistent with the 0.1 kPa response, indicating that the oil-type effect is not limited to low stress conditions. In simple terms, the higher stress level exposes differences in non-recoverable deformation tendency more clearly, so the binder that is softened the most (Olive4%) also shows the largest accumulated strain, while the mildest softening (Palm4%) shows the smallest increase. Figure 5 confirms that simulated WCO at 4% modifies the rheological response in a manner that depends on oil type, and the 3.2 kPa results indicate the need to verify rutting resistance at the mixture level using performance tests such as the Hamburg wheel tracking test.

Overall, Figure 5 confirms that simulated WCO at 4% modifies the rheological response in an oil-dependent manner. More importantly, the higher strain accumulation observed for Olive4%, particularly under 3.2 kPa, suggests a greater susceptibility to non-recoverable deformation and therefore indicates the need to verify rutting resistance at the mixture level using a performance-based rutting test. This binder-level indication is further examined in Section 3.2.5 through the Hamburg wheel tracking test.

#### 3.1.4. Frequency Sweep Test Results of Binder

**Figure 6:**
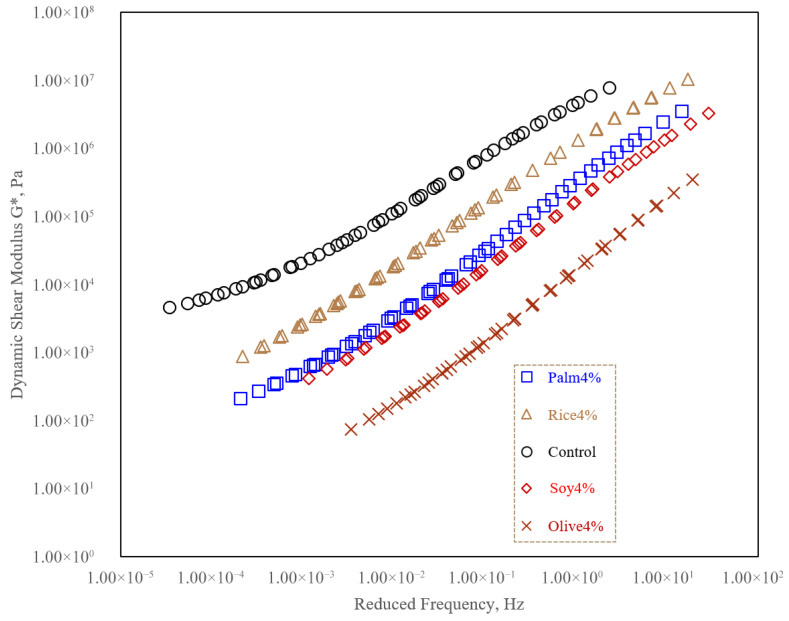
Dynamic shear modulus |G*| versus reduced frequency for Control and simulated WCO-modified binder.

Figure 6 presents the frequency sweep results expressed as the dynamic shear modulus (|G*|) plotted against reduced frequency for the control binder and the simulated WCO-modified binders (Palm4%, Rice4%, Soy4%, and Olive4%). The detailed frequency sweep analysis was limited to the 4% dosage because the preliminary binder screening results showed that 8% produced substantially stronger softening, reflected by higher penetration and lower softening point and viscosity, indicating a greater risk of over-softening. Since 4% provided the more balanced rejuvenation response, the present discussion focuses on comparing oil-source effects at this moderate dosage level.

Only the experimentally measured data points are reported, and interpretation is limited to the reduced-frequency range supported by the available test temperatures used for time–temperature superposition.

Across the investigated reduced-frequency domain, all binders show the expected monotonic increase in |G*| with increasing reduced frequency, reflecting the transition from viscous-dominated response at low reduced frequencies to a more elastic and stiffness-dominated response at higher reduced frequencies. The control binder consistently exhibits the highest |G*| throughout the measured range, indicating the stiffest overall rheological response among the evaluated binders [55].

The incorporation of WCO leads to a systematic reduction in |G*| relative to the control binder across the entire reduced-frequency range, confirming a softening and mobility-enhancing effect attributable to the oil component. Among the simulated WCO-modified binders, the stiffness ranking remains stable with no curve crossing, with Rice4% showing the highest |G*|, followed by Palm4% and Soy4%, while Olive4% exhibits the lowest |G*| over the full range. This ordering indicates that the WCO type governs the magnitude of stiffness reduction, with Olive4% producing the most pronounced decrease in |G*| and Rice4% producing the mildest reduction.

### 3.2. Asphalt Mixture Test Results

#### 3.2.1. Moisture-Conditioned ITS and TSR Results

**Figure 7:**
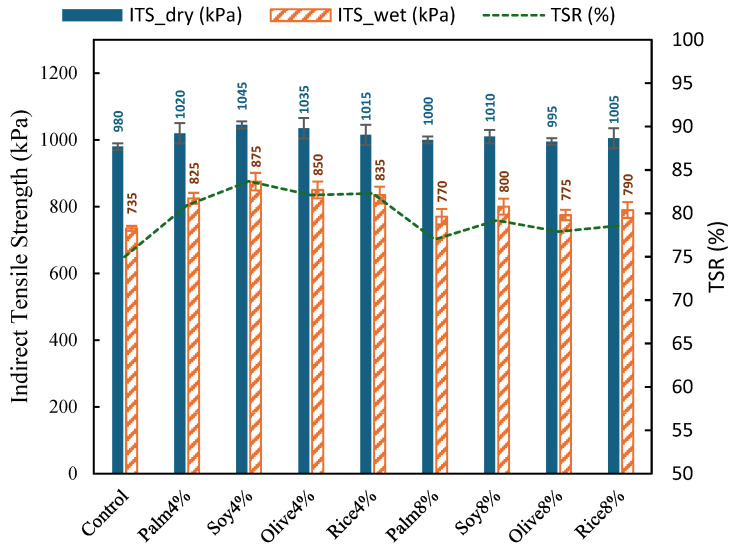
Moisture-conditioned ITS and TSR of control RAP mixture and simulated WCO mixtures (4% and 8%).

Figure 7 reports the unconditioned ITS, moisture-conditioned ITS, and TSR values for the control RAP mixture and the mixtures incorporating simulated WCO at 4% and 8%. The control mixture recorded an ITS_dry of 980 kPa and an ITS_wet of 735 kPa, giving a TSR of 75%, which indicates a noticeable strength loss after moisture conditioning.

All simulated WCO mixtures improved moisture resistance relative to the CControl, as reflected by higher ITS_wet and higher TSR. At the 4% dosage, ITS_dry increased from 1015 to 1045 kPa and ITS_wet increased more clearly from 825 to 875 kPa, resulting in TSR values of 80.9 to 83.7%. Compared with the control, this corresponds to an increase of about 12 to 19% in ITS_wet and an increase of 5.9 to 8.7 percentage points in TSR. Among the 4% mixtures, Soy4% achieved the highest moisture conditioned strength and TSR (ITS_wet 875 kPa, TSR 83.7%), followed by Rice4% and Olive4% (TSR 82.3% and 82.1%, respectively), while Palm4% remained the lowest within the 4% group (TSR 80.9%).

When the dosage increased to 8%, the improvement relative to the CCControl remained, but the performance was lower than that of the 4% group. ITS_wet values ranged from 770 to 800 kPa, and TSR values ranged from 77.0 to 79.2%. Relative to the control, TSR increased by 2.0 to 4.2 percentage points, but compared with the 4% mixtures, it decreased by roughly 2 to 6 percentage points, indicating that excessive oil dosage may reduce moisture durability gains even though it still provides some benefit over the untreated control. Within the 8% group, Soy8% again produced the highest TSR (79.2%), while Palm8% produced the lowest (77.0%).

The moisture-conditioned ITS and TSR results indicate a clear dosage effect, where the 4% simulated WCO dosage provides the most favorable balance for moisture resistance in the RAP mixture system, while 8% remains improved compared with the Control but shows reduced TSR relative to 4%. The improved ITS and TSR values suggest that simulated WCO partially restored the aged RAP binder, making it less brittle and improving its ability to coat and bond the aggregate skeleton. This produced a more continuous binder film and better internal cohesion, which increased dry tensile strength and reduced moisture-induced debonding after conditioning [29]. The stronger performance at 4% compared with 8% indicates that the benefit is controlled by dosage, since excessive softening can reduce binder film stability. This trend suggests that, at a moderate dosage, simulated WCO can enhance moisture durability, likely by improving binder film continuity and aggregate coating, thereby reducing moisture-induced debonding [56].

#### 3.2.2. Cantabro Loss

**Figure 8:**
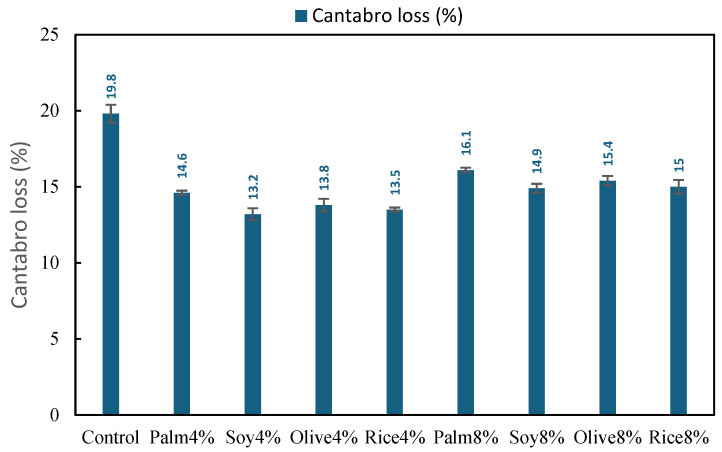
Cantabro loss of Control, RAP mixture and simulated WCO mixtures (4% and 8%).

Figure 8 summarizes the Cantabro loss results for the control mixture and the simulated WCO modified RAP mixtures. The control mixture exhibited a Cantabro loss of 19.8%, indicating comparatively higher raveling potential and weaker cohesion, which is consistent with the brittle nature typically associated with RAP-dominated mixtures without rejuvenation.

All simulated WCO mixtures reduced Cantabro loss relative to the Control, showing improved resistance to particle loss and better mixture integrity. The 4% dosage provided the largest improvement, with Cantabro loss reduced from 13.2 to 14.6%, corresponding to an absolute reduction of 5.2 to 6.6 percentage points and a relative decrease of approximately 26 to 33% compared with the control. Among the 4% mixtures, Soy4% achieved the lowest Cantabro loss (13.2%), followed by Rice4% (13.5%) and Olive4% (13.8%), while Palm4% (14.6%) remained the highest within the 4% group. This ranking suggests that, at a moderate dosage, simulated WCO improves cohesion and aggregate retention, likely by enhancing binder film continuity and reducing brittleness of the aged binder phase [57].

At the 8% dosage, Cantabro loss values remained lower than the Control but were generally higher than the 4% group, ranging from 14.9 to 16.1%. This corresponds to an improvement of 3.7 to 4.9 percentage points relative to the control (about 19 to 25% reduction), but a penalty of roughly 1.1 to 2.9 percentage points compared with the best 4% mixtures. Within the 8% group, Soy8% again performed best (14.9%), while Palm8% showed the highest loss (16.1%).

To better interpret the Cantabro loss results, they were also considered with respect to reported specification and reference limits. Previous studies and specifications have indicated that Cantabro loss values should remain within acceptable ranges to ensure adequate resistance to raveling and particle disintegration. For example, a value of 20% is often referenced based on EN 12697-17 [58], while the Malaysian JKR specification limits the maximum weight loss to 15% at 300 revolutions [59]. In addition, Babalghaith et al. [59] reported the use of Cantabro loss as an indicator of durability and cohesion performance in asphalt mixtures.

When compared with the stricter 15% maximum weight loss at 300 revolutions, clearer differences appear among the mixtures. All 4% simulated WCO mixtures satisfied this limit, with values of 14.6% (Palm4%), 13.2% (Soy4%), 13.8% (Olive4%), and 13.5% (Rice4%), indicating improved cohesion and resistance to raveling compared with the Control. For the 8% mixtures, Soy8% (14.9%) and Rice8% (15.0%) remained at or within the limit, whereas Palm8% (16.1%) and Olive8% (15.4%) exceeded it slightly. These results indicate that moderate rejuvenation at 4% provided the most favorable abrasion resistance, whereas the benefit became less consistent at 8%, likely because stronger softening reduced mixture cohesion.

#### 3.2.3. Overlay Test Results

To keep the scope practical and industry-relevant, Overlay/SCB/Hamburg testing was limited to the Control and the 4% WCO mix because 4% represents the most feasible field dosage (balancing performance gains with workability and implementation constraints) while higher dosages were treated as exploratory.

**Figure 9:**
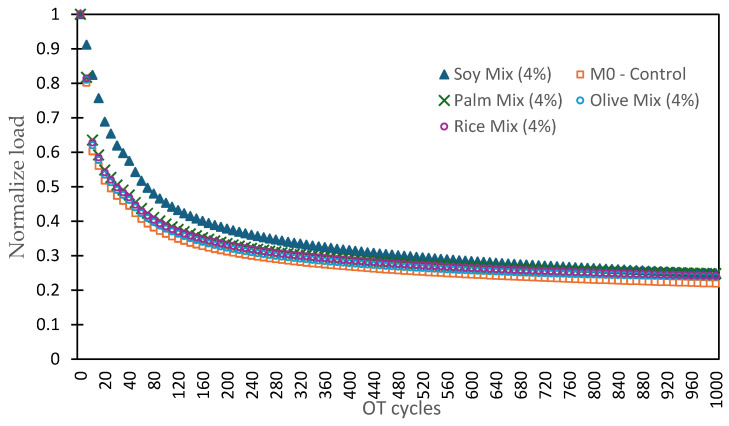
Overlay test retained load index versus cycles for Control and simulated WCO mixtures (4%).

Figure 9 presents the overlay tester response of the control mixture and the simulated WCO mixtures at 4%. The overlay tester index decreases progressively with cycle number for all mixtures, indicating accumulated cracking damage under repeated opening and closing loading [60]. In this dataset, the control mixture shows the fastest damage progression, while the simulated WCO mixtures retain higher index values throughout the test, indicating improved crack resistance after rejuvenation.

The same trend is maintained at long cycles. At Cycle 1000, the index decreases to 0.2188 for the control mixture, while the simulated WCO mixtures remain higher, reaching 0.2387 (Olive4%), 0.2412 (Rice4%), 0.2477 (Palm4%), and 0.2489 (Soy4%). Compared with the control at Cycle 1000, Soy4% retains an index that is higher by about 14%, confirming improved cracking tolerance over the full loading history.

These results are consistent with the broader mixture performance trends, where the simulated WCO mixtures also showed higher moisture-conditioned strength and reduced Cantabro loss compared with the Control.

At the early stage, where crack initiation dominates, the separation among mixtures is clear. At Cycle 40, the index of the control mixture decreases to 0.4442, while the simulated WCO mixtures remain higher, ranging from 0.4615 to 0.5749. Soy4% shows the best early crack resistance with an index of 0.5749, followed by Palm4% (0.4758), Rice4% (0.4690), and Olive4% (0.4615). Relative to the control, Soy4% is higher by about 29% at Cycle 40, indicating a substantially slower early damage accumulation.

#### 3.2.4. SCB Test Results and Fracture Energy Evaluation

**Figure 10:**
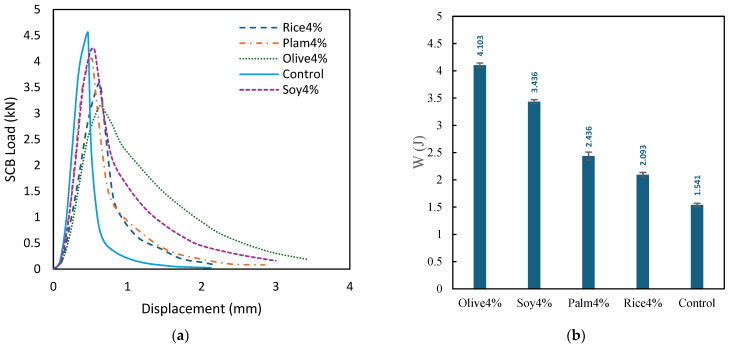
(**a**) SCB load–displacement response of the Control and simulated WCO mixtures (4%); (**b**) fracture energy (W) of the Control and simulated WCO mixtures (4%).

Figure 10a presents the SCB load–displacement responses of the control mixture and the simulated WCO mixtures at 4%. The curves show clear differences in peak load, post-peak softening, and displacement capacity, indicating that simulated WCO modifies the fracture response of the RAP mixture rather than shifting all mixtures in the same direction.

The control mixture exhibits the highest peak load, reaching 4.545 at a relatively small displacement of 0.4675 mm, followed by a sharp post-peak drop to 2.34 by 0.51 mm and a rapidly decreasing tail that reaches 0.027 at 2.125 mm. This response indicates a strong but brittle fracture behavior, consistent with the stiff nature of untreated RAP mixtures. In contrast, the simulated WCO mixtures generally show either reduced brittleness through improved post-peak retention or increased deformation capacity, depending on oil type.

Among the simulated WCO mixtures, Soy4% shows a favorable balance between strength and ductility. It reaches a high peak load of 4.25 at 0.55 mm and maintains a comparatively stable post-peak response, retaining 3.8 at 0.60 mm and sustaining non-negligible load up to 3.0 mm (final value 0.16). Palm4% also shows a relatively high peak load (4.05 at 0.5225 mm) but with a more moderate tail, reaching 0.08 at 2.85 mm. Rice4% exhibits a lower peak load (3.58 at 0.6325 mm) and a shorter tail than Soy and Palm, decreasing to 0.08 at 2.185 mm. Olive4% shows the lowest peak load (3.15 at 0.6325 mm) but the greatest displacement capacity, sustaining load to the longest displacement range and reaching 0.18 at 3.45 mm, indicating the most ductile fracture behavior among the mixtures.

To quantify this behavior further, the fracture energy (W) was calculated from the area under the load–displacement curve, as presented in Figure 10b. The fracture energy values are 4.103 J for Olive4%, 3.436 J for Soy4%, 2.438 J for Palm4%, 2.093 J for Rice4%, and 1.541 J for the control mixture. These results confirm that the simulated WCO mixtures improved fracture tolerance relative to the untreated Control mixture. In particular, Olive4% and Soy4% showed the highest fracture energy, indicating superior energy absorption capacity before failure, even though Olive4% did not exhibit the highest peak load. This confirms that fracture behavior is governed not only by peak strength but also by post-peak resistance and displacement capacity.

These SCB results indicate that simulated WCO affects fracture behavior through a trade-off between peak strength and deformation capacity. The control mixture is peak strength dominated but brittle, whereas the simulated WCO mixtures, especially Soy and Olive, exhibit improved ductility and post-peak load retention. This behavior supports the use of fracture energy as an integrated metric, since the area under the load–displacement curve can distinguish mixtures with high peak load but rapid failure from mixtures with lower peak load but sustained post-peak resistance.

#### 3.2.5. The Hamburg Test Result

**Figure 11:**
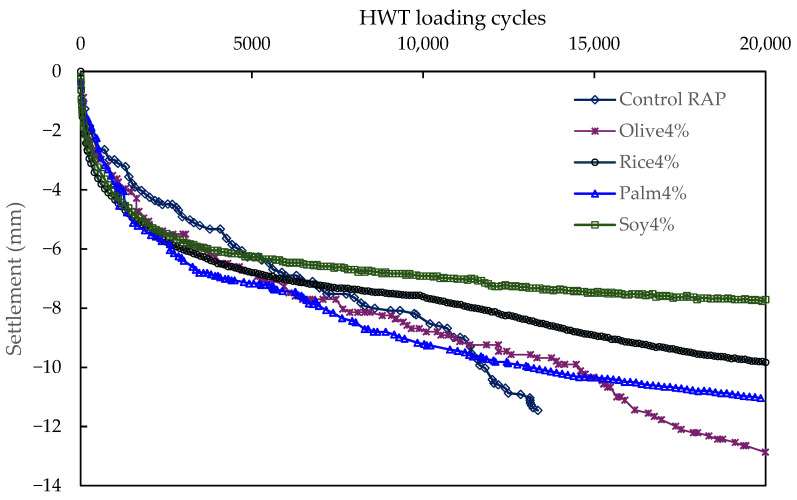
Hamburg wheel tracking rut depth versus wheel passes for control and simulated WCO mixtures (4%).

Figure 11 shows the HWT settlement development of the control RAP mixture and the simulated WCO mixtures under submerged loading up to 20,000 cycles. All mixtures exhibit the typical two-stage response, with a rapid early settlement phase (post-compaction and densification) followed by a slower accumulation phase. Clear differences in long-term rutting durability are observed among mixtures.

Overall, Soy4% provides the best rutting resistance and the most stable curve throughout the test. Its settlement remains the smallest at long cycles, ending at approximately 7.7 to 7.8 mm by 20,000 cycles (for example, the final value in your table is about 7.71 mm). Rice4% shows intermediate performance, ending at approximately 9.8 to 10.0 mm at 20,000 cycles (your table indicates about 9.83 mm), while Palm4% accumulates a larger settlement and finishes around 11 mm. Olive4% shows the poorest rutting durability among the simulated WCO mixtures, reaching the largest settlement by 20,000 cycles, approximately 12.5 to 13 mm, and its curve exhibits a clear late-stage steepening compared with the other simulated WCO mixtures. The Control RAP mixture displays an abrupt increase in settlement at mid to late cycles and the curve terminates early in the plot, which is consistent with severe damage development under Hamburg loading and highlights the vulnerability of the untreated RAP mixture under combined moisture and repeated loading.

This ranking is consistent with the mixture level durability indicators reported earlier. Soy4% showed the highest TSR and the lowest Cantabro loss, and it also maintains the most stable Hamburg settlement progression. Olive4%, which exhibited the strongest binder softening and the highest MSCR strain accumulation at the binder level, correspondingly shows the highest settlement accumulation in Hamburg.

### 3.3. Discussion

#### 3.3.1. Overall Discussion

The test results in this research confirm that the binder-level and mixture-level results show a consistent performance chain. At the binder level, the addition of simulated WCO increased penetration and reduced softening point, dynamic viscosity, and dynamic shear modulus, confirming partial restoration of the aged RAP binder and increased molecular mobility. At moderate dosage, this rheological adjustment is beneficial because it reduces excessive brittleness, improves aggregate coating and binder film continuity, and enhances the ability of the mixture to redistribute stress. As a result, the mixtures showed higher ITS and TSR values, lower Cantabro loss, and improved Overlay and SCB responses compared with the untreated Control.

However, the rheological results also indicate that performance improvement is not governed by softening alone. The MSCR results showed that binders with stronger softening also exhibited higher stress-sensitive strain accumulation, indicating a greater tendency for non-recoverable deformation. This was reflected at the mixture level by the Hamburg wheel tracking results, where the more strongly softened binders showed larger rut depth. For example, Olive4% exhibited the lowest binder stiffness and the highest MSCR strain accumulation, and it also showed the highest rut depth among the 4% mixtures, whereas Soy4% provided a more balanced binder response and correspondingly achieved the best overall mixture performance. Therefore, the mixture behavior is governed by a balance between beneficial rejuvenation of the aged binder and excessive softening that reduces deformation resistance.

#### 3.3.2. Limitations

This study has limitations that should be acknowledged. First, only two simulated WCO dosages, 4% and 8%, were considered, and the advanced mixture-level performance tests were conducted only for the Control and 4% mixtures. This decision was made because the preliminary screening results showed that 4% provided the most balanced rejuvenation effect, whereas 8% caused stronger softening and appeared less practical for detailed performance evaluation at this stage. Therefore, the conclusions from the overlay test, SCB test, and Hamburg wheel tracking test should be interpreted primarily for the 4% dosage condition. Future work will extend these advanced tests to the 8% mixtures and additional dosage levels in order to establish a more comprehensive dosage-performance relationship and to further verify the high-dosage response.

Another limitation of this study is that the simulated WCO was prepared as a controlled laboratory surrogate to reduce source variability and to enable a consistent comparison of oil-type effects under the same thermal conditioning process. This approach is useful for isolating the influence of thermally degraded oil source, but it does not fully represent the full complexity of actual waste cooking oil generated in practice, which may be further affected by repeated frying cycles, food residues, moisture, and other contaminants. Therefore, the present findings should be interpreted within the framework of controlled simulated WCO comparison. Future work should compare these simulated oils with field-collected WCO to further verify the practical applicability of the observed trends.

## 4. Conclusions

This study demonstrated that a controlled simulated WCO approach can be used to compare rejuvenator source effects in polymer-modified RAP binders and mixtures under consistent conditions. The results confirmed that simulated WCO restored binder consistency and workability in a clear dosage-dependent manner. Relative to the recovered RAP binder, the 4% binders increased penetration from 42 to 48–52 and reduced softening point from 61.0 °C to 56.9–58.6 °C, while the 8% binders further increased penetration to 58–66 and reduced softening point to 52.9–55.6 °C, indicating stronger softening and a greater over-softening risk at the higher dosage.

A major finding is that the oil source strongly governed both binder rheology and mixture performance. Olive- and Soy-based simulated WCO produced stronger softening and higher MSCR strain accumulation than Palm-based oil, while Rice remained intermediate. This binder-level trend was reflected at the mixture level, confirming that rejuvenation effectiveness cannot be judged only from softening but must also consider deformation sensitivity and rutting resistance.

At the mixture level, the 4% dosage provided the most balanced overall performance. TSR increased from 75% for the Control to 80.9–83.7% at 4%, while Cantabro loss decreased from 19.8% to 13.2–14.6%. In contrast, the benefit at 8% became less consistent, with a TSR of 77.0–79.2% and Cantabro loss of 14.9–16.1%, indicating reduced stability gains at higher dosage.

The simulated WCO mixtures also improved cracking-related performance, but the degree of improvement depended on oil type. The work of fracture increased from about 1.54 J for the Control to 2.09–4.10 J for the simulated WCO mixtures, showing that rejuvenation improved fracture tolerance by increasing ductility and post-peak resistance. However, Hamburg wheel tracking confirmed a practical rutting trade-off: Soy4% showed the lowest rut depth (about 7.7–7.8 mm), whereas Olive4% showed the highest (about 12.5–13 mm) among the 4% mixtures.

Overall, the study shows that simulated WCO is a practical rejuvenator screening framework for polymer-modified RAP systems, but both dosage and oil source must be selected carefully. Within the conditions of this study, the 4% dosage gave the best balance between binder restoration, cracking tolerance, and rutting Control, while Soy-based simulated WCO provided the most consistent overall mixture durability.

## Figures and Tables

**Table 1:** General properties of original polymer-modified asphalt binder and recovered RAP binder from PMAB pavement (representative values).

Property	Typical Requirement (Surface PMAB)	Original PMAB	Recovered RAP Binder (from PMAB Pavement)	Assessment	Test Method
Penetration (25 °C)	50–70 (0.1 mm)	60	42	Recovered binder is stiffer than original	ASTM D5 [35]
Softening point (Ring and Ball)	≥55 °C	60	61.0	Slightly higher for recovered binder, consistent with aging	ASTM D36 [36]
Rotational viscosity (135 °C)	≤3.0 Pa·s	0.50	0.69	Recovered binder shows reduced workability	ASTM D4402 [37]
Rotational viscosity (165 °C)	–	0.23	0.31	Same trend as 135 °C	ASTM D4402 [37]

**Table 2:** Physical properties of basalt aggregates and RAP aggregate (example values).

Property	Virgin Coarse Aggregate	Virgin Fine Aggregate	RAP Aggregate	Test Method
Specific gravity (SSD)	2.85	2.80	2.78	ASTM C128 [39]
Water absorption (%)	0.9	1.3	1.0	ASTM C128 [39]
Los Angeles abrasion (%)	20	–	24	ASTM C131 [40]
Flakiness index (%)	16	–	18	BS 812 [41]
Aggregate type	Crushed basalt	Crushed basalt	Basalt (from RAP)	–

**Table 3:** Properties of oils before and after thermal conditioning (example values consistent with trends observed in this study).

Oil Type	Condition	Dynamic Viscosity at 40 °C (mPa·s)	Acid Value (mg KOH/g)
Palm	Fresh	62	0.6
Palm	Simulated WCO	78	3.1
Rice	Fresh	58	0.6
Rice	Simulated WCO	66	3.8
Soy	Fresh	54	0.7
Soy	Simulated WCO	60	4.6
Olive	Fresh	50	0.8
Olive	Simulated WCO	55	5.2

**Table 4:** Mix design.

Mix ID/Name	RAP (%)	Batch Mass (kg)	RAP Material (kg)	Virgin Aggregates (kg)	Total Binder 5.2% (kg)	RAP Binder in RAP (kg)	Virgin Binder Added (kg)	Oil Type	Simulated WCO (g)
Control	25	10.00	2.50	7.50	0.520	0.125	0.395		0
Palm4%	25	10.00	2.50	7.50	0.520	0.125	0.390	Palm	5
Soy4%	25	10.00	2.50	7.50	0.520	0.125	0.390	Soy	5
Olive4%	25	10.00	2.50	7.50	0.520	0.125	0.390	Olive	5
Rice4%	25	10.00	2.50	7.50	0.520	0.125	0.390	Rice	5
Palm8%	25	10.00	2.50	7.50	0.520	0.125	0.385	Palm	10
Soy8%	25	10.00	2.50	7.50	0.520	0.125	0.385	Soy	10
Olive8%	25	10.00	2.50	7.50	0.520	0.125	0.385	Olive	10
Rice8%	25	10.00	2.50	7.50	0.520	0.125	0.385	Rice	10

Note: Recovered RAP binder is used as the base binder. Simulated WCO content is expressed as wt.% relative to the recovered RAP binder mass.

## Data Availability

The data presented in this study are available within the manuscript. Additional data related to this work are available from the corresponding author upon reasonable request.
